# A Modified Consumer Inkjet for Spatiotemporal Control of Gene Expression

**DOI:** 10.1371/journal.pone.0007086

**Published:** 2009-09-18

**Authors:** Daniel J. Cohen, Roberto C. Morfino, Michel M. Maharbiz

**Affiliations:** 1 Department of Bioengineering, University of California, Berkeley, California, United States of America; 2 Department of Electrical Engineering, École Polytechnique Fédérale de Lausanne, Lausanne, Switzerland; 3 Department of Electrical Engineering and Computer Science, University of California, Berkeley, California, United States of America; Baylor College of Medicine, United States of America

## Abstract

This paper presents a low-cost inkjet dosing system capable of continuous, two-dimensional spatiotemporal regulation of gene expression via delivery of diffusible regulators to a custom-mounted gel culture of *E. coli*. A consumer-grade, inkjet printer was adapted for chemical printing; *E. coli* cultures were grown on 750 µm thick agar embedded in micro-wells machined into commercial compact discs. Spatio-temporal regulation of the *lac* operon was demonstrated via the printing of patterns of lactose and glucose directly into the cultures; X-Gal blue patterns were used for visual feedback. We demonstrate how the bistable nature of the *lac* operon's feedback, when perturbed by patterning lactose (inducer) and glucose (inhibitor), can lead to coordination of cell expression patterns across a field in ways that mimic motifs seen in developmental biology. Examples of this include sharp boundaries and the generation of traveling waves of mRNA expression. To our knowledge, this is the first demonstration of reaction-diffusion effects in the well-studied *lac* operon. A finite element reaction-diffusion model of the *lac* operon is also presented which predicts pattern formation with good fidelity.

## Introduction

The development of methods that introduce spatiotemporal perturbations into developing, multi-cellular systems via soluble molecules has a long history [1]–[4] and a rich, recent body of literature. Specifically, advances in microfluidics [5]–[10] and biochemistry [11]–[14] are beginning to open the door to direct modulation of developmental pattern formation at the spatial and temporal scale of the cell's control circuitry. Such devices can provide spatially rich, real-time input-output (I/O) signals to bias toward developing cells into specific phenotypes. In the context of synthetic biology, such interfaces would add a degree of control over the pattern formation dynamics in multi-cellular structures that are expressing genetic circuits intended to coordinate activity through soluble molecules [15]. Initial efforts in building synthetic multicellular constructs have already begun [15], [16] and, as these mature, a robust chemical interface will be invaluable in addressing and biasing the development of patterns. In the context of control theory, such devices would allow an exploration of equilibria, stability criteria, and the non-linear dynamics of regulatory circuits. In the context of regenerative medicine and tissue engineering, these devices could potentially provide active, spatiotemporal control of morphogenesis [8], [17], [18].

While it is clear that these applications call for systems capable of high-resolution dosing of multiple chemicals onto ensembles of cells, it is less clear how best to achieve this in a way that is low-cost, versatile, and open-source. Although a number of microfluidics-based attempts have been published [5], [9], [19], [20], all have limitations in resolution, complexity of fabrication, or ease of use. As an alternative to microfluidics, we considered inkjet technology. To date, inkjets have been incorporated into a variety of biological techniques including: direct cell printing for patterning [21], [22] and tissue engineering [23]–[27], assorted cell factor printing to regulate cell positioning and behavior [27]–[30], and DNA micro-array fabrication [31]. This list demonstrates the versatility of the platform, although inkjets have yet to be used for active regulation of cellular behavior. Commercialized research-grade inkjet systems such as the Fujifilm Dimatix system exist but cost orders of magnitude more than consumer-grade printers and usually only print one ink at a time.

**Figure 1:**
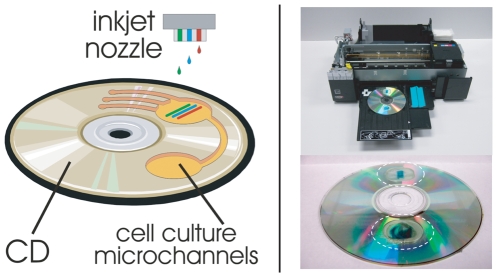
Overview of the printing system. (left) The dosing method is based on the delivery of multiple chemical compounds from piezoelectric printer heads onto specially prepared compact disk (CD) templates modified so as to support thin layers of microbial agar cultures, (upper-right) the printer used was the Epson R280, here shown being loaded with a modified CD, (lower-right) close-up of a modified CD with stand-offs for the print rollers and two LB/X-Gal agar cultures of *E. coli* dosed with lactose patterns (which induce the characteristic X-Gal blue color, see text).

This paper presents the adaptation of a commercial, low-cost, piezoelectric inkjet printer and commercial compact discs (CDs) for use as a chemical interface system designed to actively regulate cellular development (Figure 1). The printer is capable of addressing up to six different soluble chemicals and subsequently delivering precise doses of these chemicals to cell cultures at 226 dots/mm. The platform can be integrated with inline microscopy to acquire data at specific time points post-dosing. Additionally, no custom software is required for our approach, making the whole system simple and user-friendly. While the CD platform is compatible with the rich toolset of polymer microfluidics [32], [33] and could be adapted into a more sophisticated device in future work, its use here was solely as a convenient, readily modifiable substrate that was compatible with the printer. In this study we used the inkjet to control the spatiotemporal reaction-diffusion dynamics of gene expression in the *lac* regulatory system by printing specific patterns of lactose and glucose onto a field of *E. coli*.

The choice of *lac* was deliberate, in that it is commonly used in synthetic biology and has a number of interesting control features, including well-characterized feedback and multiple stable states [34]–[36]. We were surprised by the observation that the bistable nature of the *lac* operon's feedback system, when perturbed by patterns of lactose (inducer) and glucose (inhibitor), can lead to coordination of cell expression patterns across a field in ways that mimic motifs seen in developmental biology. Examples of this behavior include sharp gene expression boundaries and the generation of traveling waves of mRNA expression from single ‘trigger’ patterns. In this context, lactose and glucose are analogous to morphogens in a developing system, with the *lac* operon acting as a general template for exploring how spatially-graded perturbations generate rich behavior in bistable circuits. This is especially interesting given that *lac*, while employing positive and negative feedback and diffusible molecules (i.e. glucose and lactose), is not usually considered a reaction-diffusion system capable of generating pattern. By contrast, there is a vast literature on Turing-type and other reaction-diffusion systems and their applications to biological pattern formation [37].

## Methods

### Theory and Model

**Figure 2:**
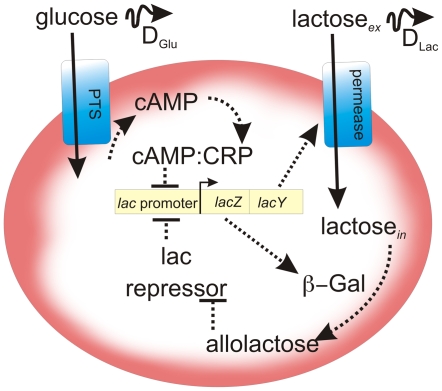
Graphical representation of the *lac* operon. This depicts the model used with the simulation. See *Appendix S1* for modeling and simulation details.

The *lac* operon is one of the best studied regulatory pathways in microbes [34]–[36], [38]. In *E. coli*, kinetic data for the entire *lac* operon is available and robust models have been developed [34], [35]. Moreover, the dynamics of the system are well understood, and are known to exhibit multiple stable points [34]–[36]. In the canonical *lac* system (Figure 2), extra-cellular lactose is taken up by lactose permease, where it is converted to allolactose by β-galactosidase. Allolactose upregulates the production of both β-galactosidase and lactose permease by inhibiting the repressor of the *lac* promoter. This lactose-based, positive feedback loop has been shown to be bistable [34]; below a certain lactose threshold, little lactose is converted while, above this threshold, the system jumps to a much higher consumption rate. Glucose acts to inhibit the conversion of lactose by lowering the transcription rate of the *lac* operon via cAMP and the catabolite repressor protein (CRP). This acts as negative feedback for the conversion of lactose. Both lactose and glucose are soluble in the extra-cellular space. We chose to model (Figure 2) the *lac* system as a set of coupled differential equations, following models developed by [34], [35] (see *Appendix S1*). The well-known X-Gal assay [39] introduces the soluble X-Gal compound, which is cleaved by β-galactosidase into 5-bromo-4-chloro-3-hydroxyindole, in turn oxidizing to 5,5′-dibromo-4,4′-dichloro-indigo, finally resulting in an insoluble blue product. This allows for β-galactosidase activity to be assayed.

### Experimental overview

All experiments involved a series of four steps, detailed below. First, bacteria were cultured in advance of an inkjet print run. Once at the proper optical density (OD_600_), they were spread over an agar substrate containing X-Gal and incubated until printing time. During the incubation period, the printer was sterilized and loaded with lactose and glucose. At the appropriate time, the bacteria were removed from the incubator, loaded onto the substrate CD, printed upon, and returned to the incubator. Additional information is provided in *Appendix S1*.

### Cell culture

*E. coli* strain MG1655Z1 was used in this study [40]; the strain contained a low-copy number plasmid (pNS2-σVL) with three reporters, one of which is GFP driven by the *lac* promoter. The initial culture, suspended in LB Agar (Sigma LB at 20 g/L and Sigma Bacteriological Agar at 14 g/L), was prepared for freezing with the addition of 50% glycerol. Colony plates were created by taking a sterile loop and transferring a sample from the freezer stock to 5 mL of LB Broth (Sigma, 20 g/L) containing *Kanamycin* (50 µg/mL). This culture was incubated at 37°C for 12 hours and then spread, via sterile probe, onto the surface of an LB Agar plate containing *Kanamycin* (50 µg/mL). The colony plate was incubated at 37°C for a further 12 hours, before being transferred to a refrigerator maintained at 4°C.

### Print medium preparation

LB Agar was poured to a depth of 750 microns in Petri dishes and chilled overnight at 4°C. Prior to plating the cells, the plates were removed from the refrigerator, and 200 µL of X-Gal solution (Sigma, 20 mg/ml in DMSO) was bead-spread onto the surface of each plate. Ten minutes were allowed for the X-Gal to penetrate the agar and for the DMSO to evaporate. Finally, 150 µL of an overnight *E. coli* suspension (diluted to OD_600_ 0.8) was bead-spread onto the surface and allowed to sit for 10 minutes, after which the plates were wrapped with Parafilm and placed in a 37°C incubator for 3.5 hours, at which point they had just entered the exponential phase [41].

### Inkjet modification and printing

As detailed descriptions of the modifications made to the Epson R280 are included in the *Appendix S1* and *Movie S1*, an abbreviated summary is presented here. The necessary modifications require commonly available tools and several hours to complete, and the procedure should be adaptable to a number of different printer types.

We used an Epson R280 inkjet for several reasons. Epson printers use piezoelectric print-heads, as opposed to thermal jet heads. While both types of print-head would probably suffice for our experiments, the mechanical nature of piezoelectric heads means that they can safely print a greater array of chemicals, and they do not impose temperature fluctuations on the printed fluid. Additionally, the R280 has the ability to print on rigid substrates (compact discs), which is not a common feature. Finally, the whole system is low cost (∼▒100) and widely available.

There are three fundamental challenges related to manipulating a printer: loading customized inks, uniquely specifying which inks are used during a print job, and interfacing with the biological substrate. Using an Epson R280 printer, we were able to load our lactose and glucose inks by interrupting the ink-charging process and manually injecting, by syringe pump, our solutions into specific color reservoirs (300 mM lactose, 500 mM glucose). This technique requires no manipulation of the ink cartridges themselves; we inject ink downstream of the cartridge, meaning that the printer functions completely normally but prints the injected solutions rather than ink. This is simpler, less prone to damage of the printer, and does not require the use of third-party hardware. Having primed the printer, the final step was to prepare it to accept a cell-bearing substrate.

We took advantage of the R280's ability to print directly onto the surface of compact discs and milled 800-micron deep wells directly into the surface of CDs. The size, geometry, and position of these wells were selected so as not to interfere with any of the printer's mechanisms (feed rollers or carriage drive system). By using a CD template in Adobe Photoshop, it was possible to create any planar pattern, uniquely specify the inks to be used, and print directly into the wells.

### Printing of chemicals onto cultured E. coli

We used sterile shim-stock to cut out individual pieces of cell-bearing agar and transfer them to the appropriate wells on the surface of the CD. The CD was then loaded into the printer, and the print job sent. No run lasted longer than two minutes, and at no point did the cells come into contact with any components of the printer, which had previously been sterilized with 70% ethanol. Post-printing, the agar slices were transferred to hydrated Petri-dishes, placed in the incubator, and observed over a period of 15 hours.

### Microscopy and image analysis

Static images were taken at 15 hours post-printing and were captured using a fluorescent backlight and an 8 mega-pixel digital camera (Canon A590). Time-lapse data was collected using an Intel QX3 microscope positioned within the incubator. All images were subsequently grey-scaled, and intensity profiles were calculated using ImageJ. The resulting intensity profiles were normalized so that higher intensity values imply a greater transmittance of light. All simulation data was obtained according to the parameter set specified in *Appendix S1*.

## Results and Discussion

There were three key goals for this work. First, we aimed to demonstrate the feasibility of using a commercial inkjet printer as a micro-dosing chemical interface for cellular systems. Second, we wished to determine whether inclusion of diffusion terms into a partial differential equation (PDE) model of the *lac* operon would predict the gene expression patterns generated by the printer. Lastly, we hoped to explore the types of morphogenetic-like behaviors that could be induced solely through direct, chemical manipulation of the *lac* operon.

### The bistability of the lac operon generates sharp gene expression boundaries

**Figure 3:**
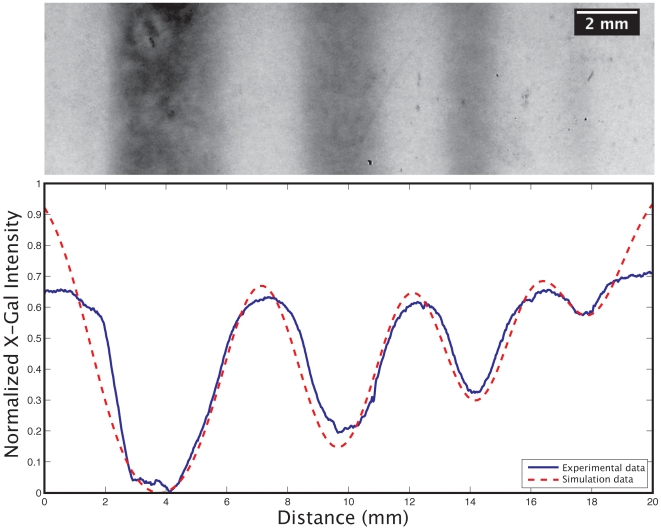
Resolution test varying only the width of the printed region. Four bars of lactose were printed with the following widths (left to right): 3.5 mm, 2.0 mm, 1.5 mm, and 0.75 mm. Note the abrupt transition to a low level of induction at the 0.75 mm bar. Further note the close agreement between the empirical data and the simulation. The discrepancies at the boundaries are a result of the optical properties of the agar at the boundaries of the sample that were not taken into account in the simulation.

**Figure 4:**
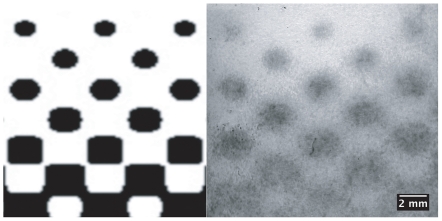
Half-toning demonstration of minimum feature spacing. Half-toning was used to produce a 2-D, graded template (left) with the feature density decreasing towards the top of the pattern. As expected, large, closely situated features tended to blur (right), while distinct features emerged when the feature density decreased. This behavior can be used to modulate feature interaction as a function of geometry and the transport properties of the medium.

We first characterized the resolution and pattern-formation capability of the printer system. As we were printing into hydrated agar (which would allow for diffusion of any dosed molecule), we could not rely on the resolution specifications of the print head. Concentrated lactose (300 mM) was printed in parallel bars of varying widths onto samples and the resulting X-Gal pattern was recorded (Figure 3). By fitting this data (in addition to the transient data presented below) to our finite element reaction-diffusion model, the effective diffusion rates were calculated. The fact that competition between diffusion and reaction rates biases the bistable response of the *lac* operon is evident in Figure 3. Below a certain width of printed inducer, diffusion reduces the peak concentration, and the lac operon never switches to its ON state (note how the fourth bar shows a marked, non-linear decrease in induction). For 300 mM lactose and our agar formulation, features smaller than 700 µm tended not to visibly induce. Thus, by varying the diffusion constant of the medium and the concentration of dosed inducer, the exact minimum width of an induced feature can be precisely controlled. A demonstration of this involves using half-toning to produce size-graded, two-dimensional features across a field of cells (Figure 4). Here, we see blurring between closely spaced, large features, but more well defined smaller features. The implication is that, by taking the transport characteristics of a system into account, we can modulate how features interact with each other.

**Figure 5:**
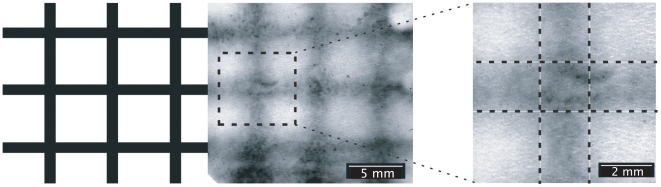
Cross-hatched lactose resolution testing. The printer template and corresponding induction profile are shown (left), alongside a close-up of a junction which demonstrates the sharp drop-off in induction that occurs, despite diffusion.

Bearing this in mind, Figure 5 shows a cross-hatched pattern used to test the uniformity of response (and resolution) across a large field of cells. This also demonstrates how the inkjet, in conjunction with a bistable circuit, can be readily used to produce sharp boundaries enclosing non-induced material even in the presence of an inter-diffusion zone. By taking advantage of bistability in the presence of weak gradients, we can achieve fairly sharp boundaries (see below), a motif observed in embryonic developmental programs [42]. Given a well-tuned simulation tool, it is possible to design and print almost any induction pattern within the resolution constraint given above.

### Inline microscopy can be used to acquire time-lapse pattern formation data

**Figure 6:**
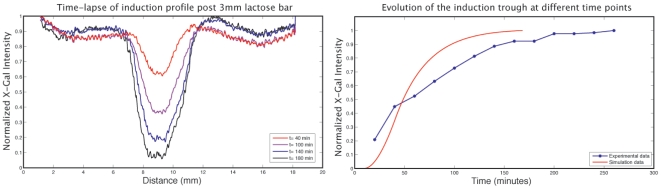
Transient lactose induction profiles. A single bar of lactose was printed and observed for 3 hrs. Induction profiles taken at various time points are presented (left) alongside the corresponding rate curves. Each data point in the empirical rate curve comes from averaging the intensity across the trough of the corresponding induction profile.

Figure 6 shows the development of X-Gal pattern over time subsequent to lactose induction. Time-lapse microscopy was performed within an incubator, with images being taken every 20 minutes for a period of 3 hours (see Methods). This data was used to fit the finite element model diffusion rates. Typically, induction becomes visible by eye after 45 minutes, and will then plateau at around 1.5 hours.

### Inkjet printing allows for the printing of 2-D spatial chemical gradients

**Figure 7:**
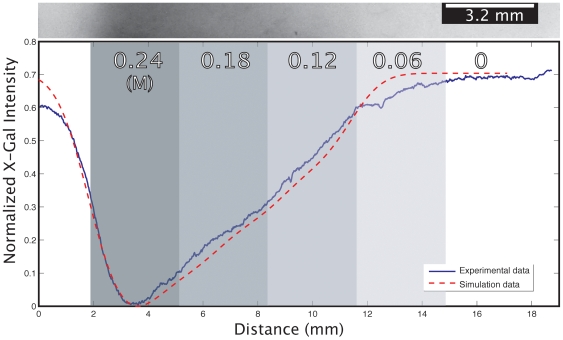
Piece-wise continuous lactose gradient profile. Here, a 5-bar (3.2 mm/bar), piece-wise continuous lactose gradient was printed, where the numbers across the bars represent the concentration of lactose printed in that bar. Again, we see very close agreement between the empirical data and the simulation.

Figure 7 shows results for a piece-wise continuous lactose gradient across a field of cells. Working from a grayscale image generated on a commercial drawing program (CorelDraw 11.0), the first bar contains 0.24 M lactose, and each subsequent bar is 20% less concentrated than the previous bar. Such a pattern is not easily attainable without a patterning device, such as the inkjet, and demonstrates the ability to produce customized, finely controlled patterns, in turn allowing fine control of cellular behavior.

### Patterns of multiple chemicals can be printed

**Figure 8:**
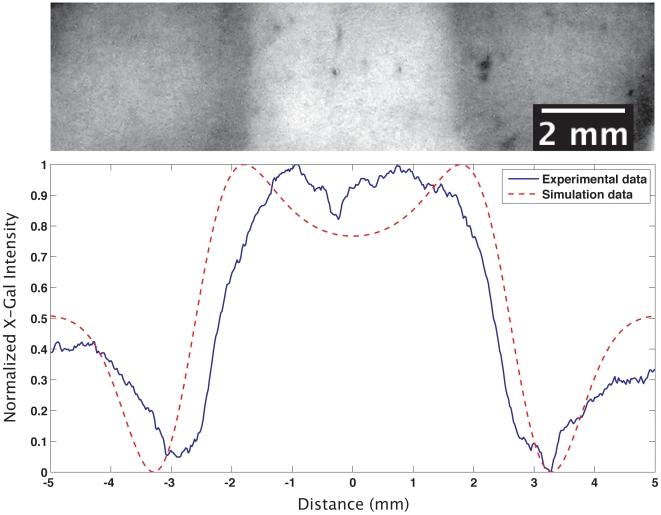
Activator-inhibitor printing with lactose and glucose. Lactose was first printed over an entire field of cells. Following this, a glucose bar, measuring 2.2 mm wide, was printed down the center of the field, on top of the lactose. Glucose is an inhibitor, while lactose is an activator. The result, with which the simulation agrees, is a region of repressed *lac* operon activity framed by dark boundaries. The increased induction arises because the lactose under the glucose is not consumed and, therefore, diffuses laterally, increasing the amount of lactose available for consumption along the boundaries.

We took advantage of the R280's ability to print multiple types of ink by creating patterns composed of both lactose and glucose. Specifically, we first printed a large, uniform field of lactose (300 mM) over an entire sample, immediately followed by a narrow bar of glucose (550 mM) printed on top of the lactose. Glucose is an exceptionally strong transcriptional inhibitor for the *lac* promoter, and this effect is demonstrated both by the complete lack of induction underneath the glucose bar, and the graded level of induction propagating out from that region (Figure 8).

### Subtle reaction-diffusion dynamics arise from patterned activator-inhibitor dosing to a bistable system

**Figure 9:**
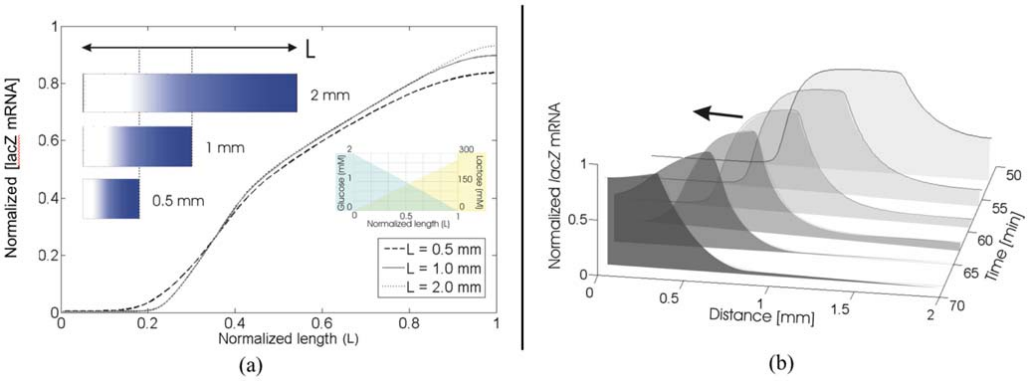
Reaction-Diffusion Dynamics. (a) Counter-gradients of activator (lactose) and inhibitor (glucose) result in scale invariant features that preserve proportions. This was demonstrated in simulation with three different trials where the lengths were varied. The simulated data shows the normalized lacZ mRNA concentration as a function of the distance for three different length cases demonstrating the phenomenon of scale invariance. (b) Plot of normalized lacZ mRNA concentration for several time steps. Here, a 2 mm long strip is simulated as having been printed with 270 mM lactose, on top of which 2 mM of glucose is printed over the region between 0 and 0.75 mm. Initially, lacZ mRNA is heavily up-regulated in the lactose-only region (everything after 0.75 mm); as both glucose is consumed for the region before 0.75 mm and lactose is consumed for the region after 0.75 mm, an mRNA peak emerges which travels to the left over time (arrow indicates direction).

In addition to predicting general features of X-Gal production patterns, our model predicts two pattern formation phenomena that arise as a result of spatial heterogeneity in the initial conditions (Figure 9).

- a)Size invariance in a printed field (Figure 9a). The multi-stable nature of the lac operon [34]–[36] allows for counter-gradients of glucose and lactose to generate an ON/OFF boundary (as in Figure 8) at the same location relative to the size of the field (Figure 9a). This is a classical motif in developmental biology known as the French Flag problem [43]; it is interesting to observe that, even in a regulatory system not intended for pattern formation, such behavior arises simply as a consequence of coupling bistable gene regulation with weak (linear) gradients.
- b)Traveling pulses of gene expression emerge from a single initial printed pattern (Figure 9b). The coupling of the *lac* operon's reactions and the diffusion of lactose and glucose lead to interesting dynamical behavior at boundaries between glucose and lactose patterns (Figure 9b). Regions dosed with lactose above the threshold for induction will immediately begin to uptake (and metabolize lactose); conversely, areas dosed with high amounts of glucose will not uptake lactose until all of the glucose is taken up. This creates a reservoir of unused lactose in the glucose-dosed regions which begins to diffuse into the lactose-dosed regions where it is taken up. This phenomenon leads to the darker regions seen at the boundaries (and predicted by simulation) in Figure 8. More interestingly, as both reaction and diffusion of glucose near the boundary deplete glucose, inhibition for lactose uptake progressively weakens and a traveling pulse of *lac* mRNA transcription arises, originating from the lactose region and extending into the glucose-rich areas.

### Conclusions

This work proposed and developed a low-cost, simple interface for regulating spatiotemporal gene expression through the use of a consumer-level inkjet printer. The system was successfully demonstrated using lactose and glucose to manipulate the *lac* operon in *E. coli*. By printing a particular chemical dosing pattern in both space and time, the printer is able to regulate the micro-chemical environments within a field of cells.

We note two fundamental biological observations from our work. First, even genetic regulatory systems not used for cell signaling (*lac*, in this case), can be adapted to mimic developmental, morphogenetic regulatory systems. As long as spatiotemporal control of chemical dosing can be achieved, many such systems might be manipulated to perform in a manner that mimics more traditional developmental pathways. Second, the ability to disturb chemical boundary conditions in space and time (especially if used in conjunction with inline imaging) may reveal new and rich dynamics even in classical gene circuits (like the canonical *lac* operon). Moreover, apart from the ability to print simple line and dot-based patterns, the printer allows for graded dosing profiles to be created. Owing to the non-linearity of reaction-diffusion systems, there are important dynamics that likely cannot be seen without graded initial conditions. Ultimately, as the system improves, the loop can be closed with real-time monitoring of genetic activity coupled to a controller that can actively correct or redirect activity by regulating the temporal aspect of the dose profiles. This system can be informed by using computational simulations as a design tool to obtain specific patterns (in much the way that Computer Aided Design systems work). Our system and techniques are simple, rapid, and inexpensive, making them an attractive option for others interested in exploring the effects of spatiotemporal dosing.

## Supporting Information

Appendix S1 Explanation of simulation methodology and procedure for modifying the inkjet.
(1.36 MB DOC)

Movie S1 Demonstration of flushing the print-head. Video details the general method for cleaning and priming the print head of the Epson R280.
(10.47 MB MOV)
