# Supplementary material for: A Modified Consumer Inkjet for Spatiotemporal Control of Gene Expression
Source: PLoS One. 2009 Sep 18;4(9):e7086. doi: 10.1371/journal.pone.0007086 (PMC2739290; doi:10.1371/journal.pone.0007086)
Supplement: Appendix S1 — Explanation of simulation methodology and procedure for modifying the inkjet. (1.36 MB DOC) [file pone.0007086.s001.doc]

**Supporting Documents**

**Simulation parameters**

|  | **Net production** |  | Degradation |  | Diffusion |
| --- | --- | --- | --- | --- | --- |
|  |  | + |  | + | -- |
|  |  | + |  | + | -- |
|  |  | + |  | + | -- |
|  |  | + | -- | + |  |
|  |  | + | -- | + |  |
|  |  | + |  | + | -- |
|  |  | + | -- | + | -- |

|  |  |  |
| --- | --- | --- |

**Table S1: Equations used in the simulation**

| **Type** | **Symbol** | **Description** | **Value** | **Unit** |
| --- | --- | --- | --- | --- |
|  |  |  |  |  |
| *Variables* |  |  |  |  |
|  | M | mRNA concentration | - | *M* |
|  | A | allolactose concentration | - | *M* |
|  | L_int_ | intracellular lactose concentration | - | *M* |
|  | L_ext_ | extracellular lactose concentration | - | *M* |
|  | G_ext_ | extracellular glucose concentration | - | *M* |
|  | cAMP | intracellular cAMP concentration | - | *M* |
|  | XgalB | X-gal Blue product concentration | - | *M* |
|  | B | β - galactosidase concentration | - | *M* |
|  | P | lactose permease concentration | - | *M* |
| *Constants* |  |  |  |  |
| Diffusion |  | External lactose | 1 x 10^-10^ |  |
|  |  | External glucose | 2 x 10^-10^ |  |
|  |  | X-Gal | 0.85 x 10^-10^ |  |
| Loss | γ_M_ | mRNA degradation | 0.411/60 |  |
|  | μ | Loss due to dilution | 0.0226/60 |  |
|  | γ_A_ | Allolactose degradation | 0.52/60 |  |
|  | γ_L_ | Lactose degradation | 0 |  |
|  | γ_P_ | Permease degradation | 0.65/60 |  |
|  | γ_B_ | β – galactosidase degradation | 8.33e-4/60 |  |
| Equilibirum | K_1_ | Repressor-allolactose reaction | 2.52 x10^-2^ |  |
|  | K | Loss rate of allolactose via conversion to glucose and galactose | 7.2 x10^3^ | -- |
|  | K_A_ | Allolactose to glucose and galactose | 1.95 |  |
| Saturation | K_L1_ | Internal lactose (L_int_) loss due to permease reversability | 1.81 |  |
|  | K_Lext_ | Lactose permease import constant | 0.26 | *mM* |
|  | K_L_ | Conversion of lactose to allolactose | 0.97 x 10^-3^ |  |
|  | Kt_Glu | Saturation of glucose transport | 15 x 10^-6^ |  |
|  |  | Fitted saturation constant for η parameter | 5 x 10^-8^ |  |
| *Time* |  |  |  |  |
|  | τ_B_ | β – gal. production via mRNA translation | 2*60[s] | *s* |
|  | τ_M_ | mRNA transcription from DNA | €0.1*60[s] | *s* |
|  | τ_P_ | Permease production via mRNA translation | 0.83*60[s] | *s* |
| *Rate* |  |  |  |  |
|  | k_B_ | Relates β – gal. production to mRNA concentration. | 0.677 | -- |
|  | k_P_ | Relates permease production to mRNA concentration | 13.94 | -- |
|  | α_L_ | Inversely proportional to L_ext_ | (2.88 x10^3^)/60 |  |
|  | α_M_ | Production rate of mRNA from DNA transcription | 9.97 x 10^-7^ |  |
|  | α_A_ | Production rate of Allolactose from lactose mediated by β – galactosidase |  |  |
|  | α_B_ | Production rate of β – galactosidase through mRNA transcription | (1.66 x10^-2^)/60 |  |
|  | α_P_ | Production rate of Permease | 10/60 |  |
|  | β_A_ | Loss rate of Allolactose via conversion to glucose and galactose | (2.15 x10^4^)/60 |  |
|  | β_L_ | Intracellular lactose loss to the extracellular fluid because of reversible nature of the permease-mediated transport | 44.166 |  |
|  | Г_0_ | Spontaneous rate of mRNA production due to a repressor not bound | 7.25 x10^-10^ |  |
|  | kt_glu | Glucose transport constant | 3000 |  |
|  | k_cAMP_ | cAMP sythesis rate constant | 1 x10^-3^ |  |
|  | K_cAMP_ | Inhibition constant for cAMP production | 4 x10^-5^ |  |
|  | *scAMP* | Composite cAMP excretion and degradation rate. | 2.1/60 |  |
| *Efficiency* |  |  |  |  |
|  | η | Efficiency parameter for mRNA production regulated by cAMP | -- | -- |
|  | η_0_ | Maximum value for efficiency parameter η | 1 | -- |

|  |  |
| --- | --- |

**Table S2: Simulation parameters**

**Development of the model and simulation**

The model for the lactose feedback (Table S1, Eqns. 1 – 4) on the *lac* operon is based on [27] with order reduction shown in [30] and with the introduction of separate variables for internal (L_int_) lactose which does not diffuse from cell to cell and external lactose (L_ext_) which is allowed to diffuse. The effect of glucose (Table S1, Eqns. 5 -7) is modeled after [28]. Glucose acts by altering the concentrations of cAMP and, in turn, the concentration of the cAMP:CRP complex (see [28] Eqn 5 and Appendix 1) whose presence enhances transcription initiation when it is bound near the *lac* promoter. This is modeled as an efficiency factor, η, which can have a value from 0 to 1. In order to determine the fraction of total binding sites occupied by the cAMP-CRP complex, a mass balance is required ([28]):

1. [CRP] = [CRP_f_] + [CRP:E] + [CRP:cAMP] + [CRP:D] + [CRP:cAMP:E] + [CRP:cAMP:D]
2. [cAMP] = [cAMP_f_] + [CRP:cAMP] + [CRP:cAMP:E] + [CRP:cAMP:D]
3. [E] = [E_f_] + [CRP:E] + [CRP:cAMP:E]

where

[CRP:cAMP] = K_ns_[CRP_f_][cAMP_f_]

[CRP:E] = K_np_[CRP_f_][E_f_]

[CRP:D] = K_nd_[CRP_f_][D]

[CRP:cAMP:E] = K_nsp_[CRP:cAMP][E_f_]

[CRP:cAMP:D] = K_nsd_[CRP:cAMP][D_f_]

here

the subscript *f* denotes the concentration of free compound

[D] is the concentration of nonspecific DNA binding sites (0.0118 M)

[E] is the CRP:cAMP DNA binding site concentration (8.47x10^-12^ mol/ g

DCW)

[CRP] is the cAMP receptor protein concentration (2 x 10^-6^ M)

K_np_ 10^6^ 1/M

K_nd_ 10^6^ 1/M

K_ns_ 2 x 10^9^ 1/M

K_nsp_ 1 x 10^9^ 1/M

K_nsd_ 1 x 10^5^ 1/M

If one assumes 50% of the non-specific DNA binding sites are unoccupied (D_f_ = D/2), then this is a system of three equations and three variables (E_f_, cAMP, CRP_f_). From the values of these three variables, we can calculate the efficiency factor, [28, Eq. 5]

(4)

To avoid having to solve these equations at every time step, we noted that a plot of cAMP vs. η follows a typical saturation curve (Figure S1):

(5)

Thus, we can calculate the efficiency as a function of cAMP using the fit equation above at a given time-step within the FEM solver (see below). Moreover, note that smaller values of K_fit_ sharpen the cAMP-dependent activation of mRNA transcription, thereby sharpening the effect of glucose on shutting off lactose metabolism. K_fit_ was one of the parameters adjusted in order to fit the lactose-glucose simulations to the data (see below).


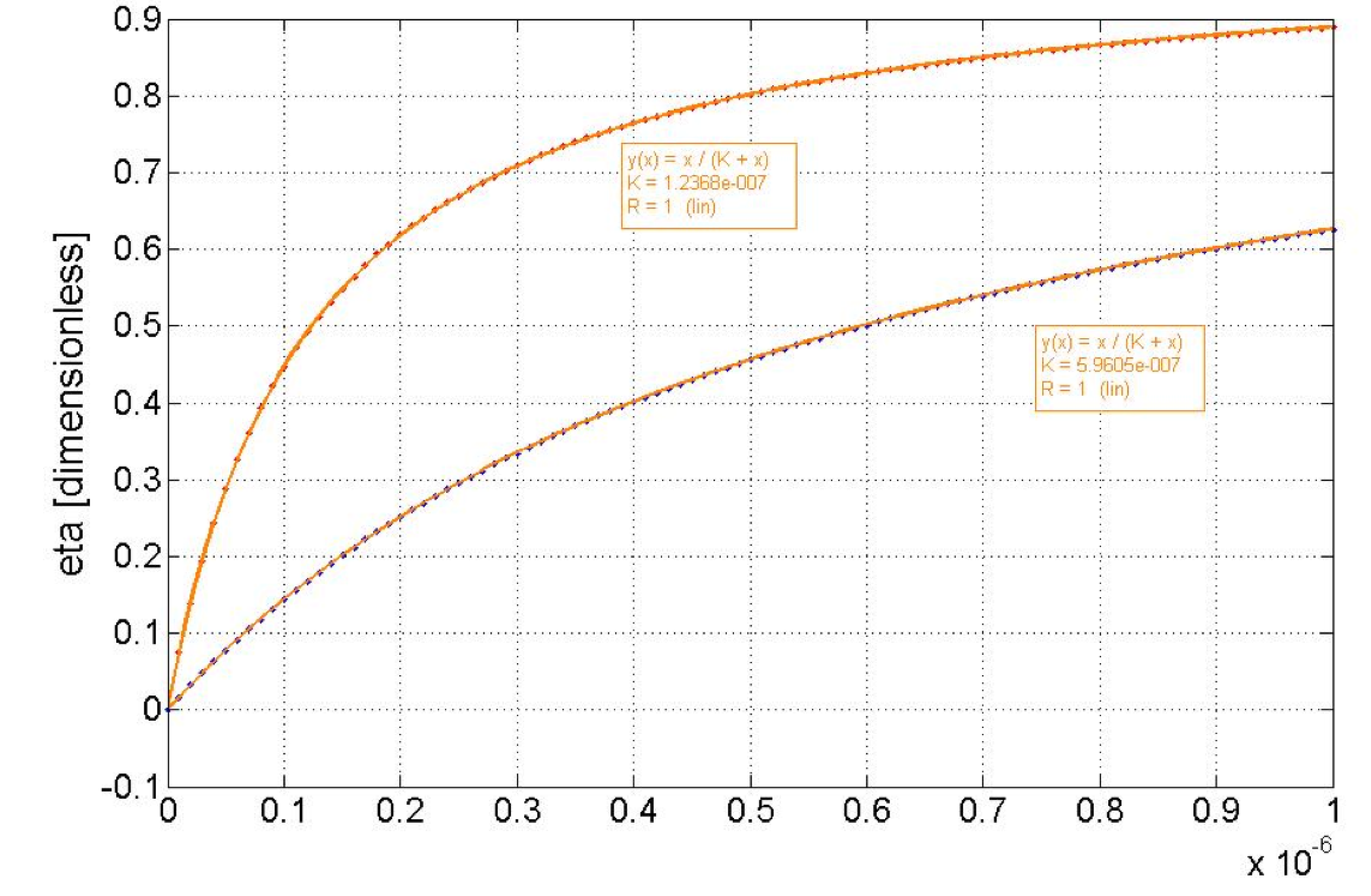


**Figure S1: cAMP vs. η saturation curve**

Blue dots plot η using Eqn. 4 and the analytical solution to the mass transfer equations (Eqns. 1 - 3); the orange line is the fit using Eqn. 5.

**Finite Element Analysis**

Finite element analysis was performed in COMSOL 3.4 using the *Stationary* and *Time Dependent* *Diffusion* modules. The reaction and diffusion terms in the equations shown in Table 1 were entered into a rectangular area matching the size of the cell growth area in the experiments. Zero flux boundary conditions were used. Initial concentrations of lactose and glucose were set in the L_ext_ and G_ext_ variables, respectively. Sharp boundaries in initial concentrations were modeled using the logistic equation to avoid infinite slopes and aid convergence. When matching data to simulation, we varied diffusion rates and K_fit_ about their nominal values to obtain the best fit to all available data (i.e. the model was made to match all data sets presented in the paper). In most cases, better fits could be obtained if each data set was fit independently, which would be expected due to uncontrolled experimental variations (e.g. local temperature effects on diffusion, local cell density variations, etc.)

**Validation**

In addition to the fact that the model fits the experimental data fairly well, we also compared to it prior analytical models of *lac* behavior and found similar agreement. In particular, we verified the characteristic bistability of *lac,* demonstrating (Figure S2) that the bistable point occurs at an external lactose concentration of 60 μM [29, 30].


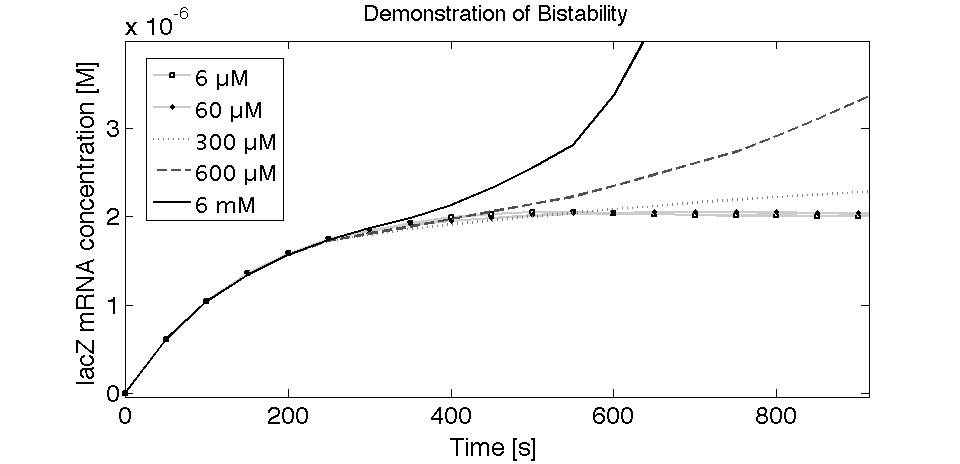


**Figure S2: Demonstration of Bistability**

Plot of lacZ mRNA concentration as a function of time for various external lactose (Le) doses. To generate this plot, an initial, homogenous concentration of lactose was applied over the simulated field and the resultant mRNA concentration was plotted over time (as the lactose was consumed). Our model exhibits the same bistability as those in [*,*]. Namely, the threshold concentration of external lactose (Le) required to trigger the 'ON' state of the lac operon is 60 uM. At or below this dose, the steady-state mRNA concentration is ~ 2uM. Above this those, the lac operon is strongly upregulated and the amount of mRNA produced is a function of the amount of lactose dosed. Once all lactose is consumed (around 3600 s), the mRNA levels return to the quiscent concentration of ~2 uM (not shown).

**Supplementary Protocol Information**

*Cell-Specific Printing Protocols*

Prior to an experiment, an overnight culture would be produced from the colony plate. A sterile loop was used to transfer a monoclonal colony to 5 mL containing 5% *Kanamycin*. This was incubated overnight for 12 hours. During the incubation period, LB Agar culture plates were produced. LB Agar (prepared as above) was autoclaved, cooled to 37 °C, inoculated with 5% *Kanamycin*, and poured into sterile, 100 mm x 100 mm square plates to a depth of 750 microns. The resulting plates were then wrapped with Parafilm and placed in a 4 °C refrigerator overnight.

At the beginning of each experiment, the overnight cultures were diluted with sterile LB to OD_600_ 0.8, and the plates were infiltrated with X-Gal (Sigma). An X-Gal solution was prepared by dissolving 20mg/ml in DMSO. Each plate received 200 μL of the X-Gal solution and bead-spreading was used to distribute this uniformly over the surface. Following this, the plates were allowed 10 minutes for the X-Gal to infiltrate the agar and the DMSO to evaporate. This procedure was implemented after it was noticed that shorter times resulted in X-Gal precipitation on the surface of the agar during the next step. After infiltration, 150 μL of *E. coli* were bead-spread onto the surface of each plate, and a further 10 minutes were allowed for stabilization, after which the plates were wrapped in two layers of Parafilm, and incubated at 37 °C for 3.5 hours. Two layers of Parafilm were found to be necessary owing to the angular geometry of square Petri dishes. A single layer tended to rupture too easily, resulting in non-uniform drying of the agar surfaces.

*Inkjet Modification*

Prior to any printing of custom inks, the printer was dissembled into its component parts, and vestigial components were removed to free-up space. This step was not strictly necessary, but it makes it simpler to sterilize the printer and manipulate samples. Specifically, the paper-feed tray, anterior-rail, anterior paper roller, top-cover, and primary paper roller were removed to allow easier access to the printing field.

The R280 was chosen for its ability to print on compact discs (CD). It does this by feeding a CD through a series of sprung, rubber rollers. These rollers apply pressure to the disc, and when they are turned the disc translates in exactly the same way that paper is normally fed through the printer. While this approach is fine for standard CDs, care must be taken to keep the rollers from coming into contact with the sample area. While it is reasonable to assume that keeping the samples below the surface of the disc will protect them from the rollers, the rollers, by virtue of being sprung, tend to pitch down into the wells as they go over them, resulting in sample contamination and deformation. To counteract this problem, 1 mm high masking tape stand-offs (see Figure 1, main text) were placed along either side of the wells and provided sufficient clearance to prevent the rollers contacting the samples.

In order to prepare the Epson R280 to print custom inks, the ink lines must first be flushed. To do this, the ink cartridges must first be removed, and flexible tubing must be connected to each of the ink-input ports. Flushing itself is accomplished with a standard syringe pump that is attached to the tubing. The lines were cleaning by flushing 10 ml of room temperature, de-ionized water through them, followed by 10 ml of 70% ethanol. This protocol was generally sufficient, and final assessment of its success was based upon whether or not any color was apparent on white filter paper held under the head during flushing. Ultimately, this process results in there being no residual ink anywhere downstream of the ink cartridges.

Once the lines were flushed, the print-head itself needed to be primed. Proper priming of a piezo print-head implies that the reservoirs downstream of the ink cartridges are filled with the desired custom inks. Priming is achieved the same way as flushing, except now each line is filled with a specific ink. In this case, the Black and Yellow lines were selected for lactose and glucose, respectively, owing to their ease of specification in software. Solutions of 1g of lactose or glucose per 10 ml of sterile DI were prepared and syringed into the Black and Yellow lines. Again, 10 ml of fluid were flushed through each line, and care was taken to ensure that no air entered the lines (this creates blockages). The remaining lines were filled with sterile DI. It is crucial to note that the priming process must take place at a very specific moment in the printer’s initiation protocol. In the case of the Epson R280, priming must be performed immediately prior to actually printing, because this is the window where the printer drivers expect that the system is fully primed and ready to print. Modifying the inks during this phase essentially tricks the system. This means the print-head must be primed while the printer is powered on and ready to print, otherwise the printer will re-initialize and flush the lines with ink.

While it is possible to clean and prime the print-head by flushing fluid through the print-head and into the printer’s internal waste collection system, this is not recommended as it can overflow the waste-ink tank. Rather, it is better to perform all flushing in a manner that keeps the excess fluid from collecting in the printer. We achieved this through a combination of absorbent towels and manually repositioning the print-head during the cleaning and priming procedures. Movie S1 presents this procedure in greater detail.

The printer can be controlled through the use of its default label-making software, or through any graphics program, such as Photoshop. Here, Photoshop was used, along with a custom template file that printed over the surface of the CD. Printing requires nothing more than drawing a pattern in the desired colors and sending the file to the printer. However, care must be taken when colors are selected. All graphics programs and printers employ default color profiles. These profiles are particular dangerous when single color resolution is required. For instance, black on a CMYK printer actually consists of varying percentages of C, M, and Y. This is done to enhance the appearance of the color on a printed page, and can be disastrous for this type of experiment. To circumvent this, customized color profiles were created to uniquely address Black and Yellow. Additionally, all non-essential colors were flushed with DI. Finally, all two-chemical experiments were performed by flushing all the lines but the lactose line, printing the first stage of the pattern, and then flushing all the lines but the glucose line. While non-rigorous experiments suggest that this is unnecessary, it acts as a failsafe in case the color addressing does not perform as expected.

The fluid volume being delivered per linear centimeter can be approximated from the technical datasheets for the printer. The volume of liquid printed ranges between 1.5 – 10 pL per drop; at the 1440 linear dpi resolution we used, this corresponds to approximately 8x10^3^ pL / cm. That said, a caveat to consider with all consumer inkjets is that they utilize proprietary droplet-placement algorithms that determine the droplet spacing, volume/droplet, and number of droplets/dot.

*Preparation of the Custom-Dishes from Compact Disks*

The R280 can natively print on the surfaces of CDs, making CD-like substrates the perfect medium for bio-printing. Rather than physically altering the spacing between the printer and the substrate to introduce standard Petri dishes (a difficult procedure), we directly machined actual compact disks, thereby requiring no complex modifications to the printer itself. Specifically, disks were placed in a milling machine and 800 micron deep wells were milled directly into the surface. The size, geometry, and position of the wells were selected so as not to interfere with the printer mechanisms (feed rollers or print-head carriage). Post-machining, the disks were cleaned of all debris and washed in ethanol—steps that were performed prior to every experiment.

*Printing of chemicals onto cultured E. coli*

After 3.5 hours of incubation, the bacteria plates were removed from the incubator and prepared for printing. We used sterile shim-stock to cut out individual pieces of cell-bearing agar and transfer them to the appropriate wells on the surface of the CD. The CD was then loaded into the printer and the print job sent. No run lasted longer than 2 minutes, and at no point did the cells come into contact with any components of the printer. Post-printing, the agar slices were transferred to hydrated Petri-dishes (containing sterile agar in their lids), placed in the incubator, and observed over 12 hours.
